# Koch’s New Treatment for Tuberculosis

**Published:** 1890-12

**Authors:** 


### Editorial.

### KOCH'S NEW TREATMENT FOR TUBERCULOSIS.

Recently the daily papers have contained many and highly sensational accounts of the wonderful results obtained with Dr. Koch's new remedy for tuberculosis. Extravagant claims as to its efficacy have been made, which must be controverted or substantiated by the accumulated experiences of many competent observers extending over a considerable period of time.

Koch, himself, has been very modest in his claims, and greatly deprecates the—as he thinks—premature wide publicity given the subject. Koch's reputation for prudence, combined with his pre-eminent abilities as a scientist, must command from the profession, at least, hopeful expectancy pending a thorough investigation of his claims and the evidences upon which they are based.

The remedy is a transparent liquid called "parataloid"—composition as yet not given to the profession—which does not tend readily to deterioration in its pure state, but which soon decomposes when sufficiently attenuated to be used with safety as an injection. The most favorable sights for the injections is the back, between the shoulders and lumbar region, because in these situations the pain and local reaction from the injections have been found to be least. The injections are followed by marked general reaction in tuberculous patients, while healthy or non-tuberculous individuals are scarcely affected at all. So it may prove of great diagnostic value in incipient tuberculosis of the lungs or other organs.

The reaction begins in about four or five hours after the injection and lasts from twelve to fifteen hours. It is accompanied by fever, increased cough, pain in the limbs, general lassitude and occasional vomiting. In pulmonary tuberculosis there is at first increase of expectoration containing bacilli in abundance, followed by decrease of expectoration and bacilli, until the sputa becomes mucus, devoid of bacilli, and in favorable cases there is entire cessation of both. Only incipient cases, or those which have not progressed to formation of large cavities, seem to be cured, the more advanced cases obtaining only temporary amelioration of the symptoms. Lupus tubercular affections of joints, glands, etc., are also amenable to the treatment. Lupus is apparently cured by the treatment, many cases by one or two injections. The bacilli seem not to be affected by the remedy, its influence being exerted upon tuberculous tissues. Sufficient time has not elapsed to verify the permanence of apparent cures.

No doubt many doctors longing for newspaper notoriety and a few with more venal motives, will make abortive and unscientific attempts to carry out the treatment. But the profession must await with patience the verdict of competent observers from all over the world who are congregating in Berlin, and who, we may hope, will soon be able to give us authentic accounts of the method of treatment and the results obtained.

In the meantime we can only hope for the consummation of the grandest of therapeutical achievements. What a field for research and discovery it would open up!

